# Central auditory deficits associated with genetic forms of peripheral deafness

**DOI:** 10.1007/s00439-021-02339-3

**Published:** 2021-08-25

**Authors:** Nicolas Michalski, Christine Petit

**Affiliations:** Institut de l’Audition, Institut Pasteur, INSERM, 75012 Paris, France

## Abstract

Since the 1990s, the study of inherited hearing disorders, mostly those detected at birth, in the prelingual period or in young adults, has led to the identification of their causal genes. The genes responsible for more than 140 isolated (non-syndromic) and about 400 syndromic forms of deafness have already been discovered. Studies of mouse models of these monogenic forms of deafness have provided considerable insight into the molecular mechanisms of hearing, particularly those involved in the development and/or physiology of the auditory sensory organ, the cochlea. In parallel, studies of these models have also made it possible to decipher the pathophysiological mechanisms underlying hearing impairment. This has led a number of laboratories to investigate the potential of gene therapy for curing these forms of deafness. Proof-of-concept has now been obtained for the treatment of several forms of deafness in mouse models, paving the way for clinical trials of cochlear gene therapy in patients in the near future. Nevertheless, peripheral deafness may also be associated with central auditory dysfunctions and may extend well beyond the auditory system itself, as a consequence of alterations to the encoded sensory inputs or involvement of the causal deafness genes in the development and/or functioning of central auditory circuits. Investigating the diversity, causes and underlying mechanisms of these central dysfunctions, the ways in which they could impede the expected benefits of hearing restoration by peripheral gene therapy, and determining how these problems could be remedied is becoming a research field in its own right. Here, we provide an overview of the current knowledge about the central deficits associated with genetic forms of deafness.

## Introduction

Hearing impairment is the most frequent sensory defect. It affects 466 million people, more than 6% of the world population. It has been estimated that, by 2050, over 700 million people will be affected by disabling hearing disorders [defined as a hearing impairment of more than 35 decibels (dB) in the ear with the best hearing] (WHO 2021). The incidence of age-related hearing loss, or presbycusis, which affects a quarter of the world population over 60 years of age, will continue to increase, due to the ageing of the global population and the increasing number of people overexposed to noise in heavily urbanised and industrialised areas (WHO 2021).

It has been estimated that 60–80% of congenital or prelingual forms of deafness in high-income countries are of genetic origin (Shearer et al. 1993; Koffler et al. 2015). Most of these genetic forms are monogenic, DFNB (autosomal recessive) forms, generally causing severe or profound deafness. The remaining forms are mostly DFNA (autosomal dominant) forms, which are generally associated with less severe, progressive hearing impairment. Most of the genes yet to be identified underlie very rare forms, but the rate of discovery of genes responsible for deafness remains high, thanks to next-generation sequencing (NGS) technologies. The causal genes for 140 isolated (non-syndromic) and 400 syndromic forms of deafness with onsets from birth to early adulthood have been identified (Noman et al. 2020; Van Camp and Smith 2021). According to a recent report by the International Mouse Phenotyping Consortium, 52 previously unknown candidate deafness genes have been found in the 3006 knockout mutant mouse strains analysed to date. Extrapolation of this number to the whole genome suggests that about 350–400 additional genes responsible for monogenic forms of hearing impairment may remain to be discovered (Bowl et al. 2017).

The vast majority of these genetic forms of deafness involve defects of the cochlea, the auditory sensory organ. Their extensive study through mouse models has been key to characterisation of the molecular mechanisms underlying the normal development and functioning of the peripheral auditory system (the cochlea and its afferent innervation), revealing crucial roles for structures that were originally underappreciated, such as the top connectors of hair cell stereocilia (Verpy et al. 2008). These studies have also elucidated the molecular networks and protein complexes involved in key cochlear functions (Richardson et al. 2011; Richardson and Petit 2019; Corey et al. 2019), thereby allowing the parallel elucidation of the pathogenesis of various deafness forms. Over the last 10 years, this work has naturally led to the emergence of gene therapy as a potentially promising treatment for hearing impairment of genetic origin, with the establishment of several proofs of concept in mouse models of human hereditary deafness (Akil et al. 2012, 2019; Emptoz et al. 2017; Wu et al. 2021). Studies reporting hearing restoration in mouse models following gene therapy interventions from P20 onwards are of particular interest, because they suggest that postnatal gene therapy interventions in humans should be effective, based on the comparative development of hearing in mice and humans. Hearing begins on P12 in mice, whereas hearing onset occurs in the 19th week of gestation in humans. The degree of maturation of the mouse central auditory system on P20 is thought to be that at an age of one to two years in humans (Wang et al. 2020; Knipper et al. 2020). However, a full restoration of auditory thresholds by interventions from P20 onwards has been achieved for very few genetic forms of deafness to date (Akil et al. 2019).

**Fig. 1:**
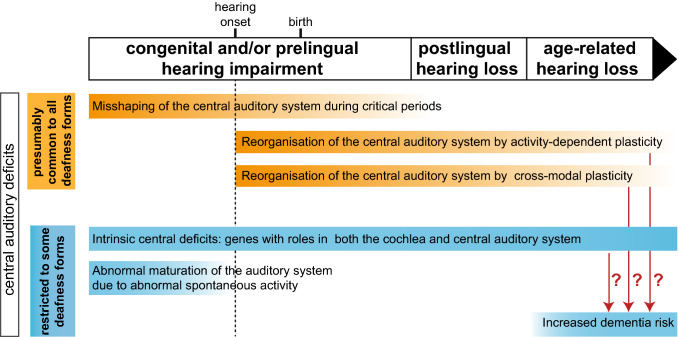
Central auditory deficits associated with different types of genetic forms of peripheral deafness in humans. The central auditory deficits shown in orange constitute an ensemble of indirect effects presumably common to all forms of congenital peripheral deafness. Other central deficits in blue denote deficits that may be combined in specific genetic forms of congenital deafness. With the exception of genes playing intrinsic roles in both the central and peripheral auditory systems, all the central auditory deficits described here are indirect consequences of peripheral hearing impairment. The time scale is based on the development of the human auditory system

In addition to peripheral deficits, these genetic forms of deafness also affect the central auditory system. Indeed, at any age, complete or partial auditory deprivation of peripheral origin (whether caused by deficits of the outer, middle or inner ear) has indirect deleterious effects on the development and morphofunctional organisation of the central auditory system. Hearing impairment in children delays the acquisition of speech and language, and may, thus, affect cognitive development and may lead to social isolation (Shojaei et al. 2016). Following auditory deprivation, several forms of plasticity come into play, leading to a reorganisation of the central auditory circuits (Fig. 1). In particular, congenital or early forms of profound deafness, whether of genetic or non-genetic origin, prevent the normal shaping of the central auditory circuits induced by sound exposure during the early phases of high plasticity for brain development (de Villers-Sidani and Merzenich 2011; Schreiner and Polley 2014; King et al. 2018; Kral et al. 2019; Glennon et al. 2020). Such alterations may even occur in cases of mild hearing loss, in which they are likely to be underdiagnosed. For example, heterozygous carriers of mutations in *USH2A* encoding usherin have been shown to have a higher risk of altered low-frequency sound perception associated with a developmental language disorder although they are generally considered to be unaffected carriers (Perrino et al. 2020). Moreover, increasing numbers of proteins encoded by causal genes of peripheral deafness are being found to have direct or intrinsic roles in the central auditory system (their defects thus lead to intrinsic central auditory deficits) (Fig. 1). Studies of the corresponding animal models have revealed that intrinsic central auditory deficits may coexist with peripheral deficits, as in mouse mutants for the microRNA miR-96, which operates in both the peripheral auditory system and some hindbrain auditory nuclei (Lewis et al. 2009; Mencía et al. 2009; Friedman and Avraham 2009; Schlüter et al. 2018). Both indirect and direct (intrinsic) central auditory deficits are often masked by peripheral defects and, therefore, go unnoticed in the absence of a systematic exploration, which requires a residual hearing.

These central auditory alterations or deficits are problematic in several ways. They interfere with the outcome of hearing rehabilitation achievable with cochlear implants—neural prostheses that bypass the outer and middle ear, and the defective cochlea to stimulate the primary auditory neurons forming the spiral ganglion nerve directly. They are also likely to limit the hearing restoration expected from future auditory peripheral gene therapy interventions. This is why the recovery of auditory perception at the cortical level is beginning to be taken into account in animal studies developing new gene therapy treatments (Nist-Lund et al. 2019). In addition, the impact of central auditory deficits extends well beyond the auditory system itself. Peripheral hearing loss also affects cognitive functions. Mid-life hearing loss of peripheral origin is the leading potentially modifiable risk factor for dementia with a population attributable fraction of 9% (Livingston et al. 2017) (Fig. 1). In addition, associations between dysfunctions of the auditory system and severe psychiatric disorders, such as schizophrenia—a complex psychiatric disorder with neurocognitive function deficits—have also been reported (Linszen et al. 2016) but the underlying mechanisms linking hearing impairment and schizophrenia remain poorly understood. Notably, *Df1*/ + mutant mice—a model of the 22q11.2 deletion in humans, the strongest genetic risk for schizophrenia (Paylor and Lindsay 2006)—are prone to hearing loss (60% of mice), which is correlated with a higher susceptibility to middle ear infections due to haploinsufficiency of the *Tbx1* transcription factor gene. In these mice, hearing loss was found to promote schizophrenia-relevant brain and behavioural abnormalities, including altered electrophysiological measurements of central auditory gain, and was associated with a smaller number of parvalbumin inhibitory neurons in the auditory cortex than were found in *Df1*/ + mutant mice with no hearing loss (Zinnamon et al. 2019).

In the framework of this special issue on ‘The Molecular Genetics of Hearing and Deafness’, we focus on the central auditory deficits associated with peripheral auditory deficits of genetic origin. We discuss the heterogeneity of these defects and highlight the importance of deepening our understanding of the underlying mechanisms (see Fig. 1), to optimise the development of gene therapy for deafness.

## Central auditory deficits in congenital and prelingual genetic forms of deafness

Several forms of plasticity operating in different brain structures, dealing with the processing of different sound attributes, of variable strength during the individual’s lifetime, shape auditory brain microcircuits, ensuring their adaptability and optimisation in changing environments (de Villers-Sidani and Merzenich 2011; Kral 2013; Schreiner and Polley 2014; Kral et al. 2019; Glennon et al. 2020). However, plasticity may become detrimental in some pathological conditions, such as auditory deprivation (Fig. 1). Furthermore, by allowing a misshaping of the structure and function of neuronal networks, such plasticity could in the long term make hearing rehabilitation even more challenging. In early life, the primary auditory cortex sensory areas pass through critical periods, windows of enhanced plasticity opening sequentially for the various features of sounds (Reh et al. 2020). For example, the continuous exposure of rat pups to short pulses of white noise (50 ms noise pulses presented 6 times per second at 65 dB) for 20 days starting on P9, i.e. a period comprising hearing onset (about P12 in rats) and the phase of increasing cochlear sensitivity (from P12 to P15), affects the maturation of the frequency map in the primary auditory cortex (Zhang et al. 2002), whereas the same exposure starting on P30 has no such effect. By contrast, the critical periods for more complex sound features, such as frequency-modulated sweeps, which are the key to understanding speech and appreciating music in humans, have a later onset, at P32, and come to an end at P38 in mice (Bhumika et al. 2020). This succession of plasticity windows is apparent in humans through the successive maturation of the corresponding auditory perceptual skills; accurate discrimination of frequency matures early, before the age of one year, whereas the discrimination of frequency modulation does not mature fully until about the age of 10 years (Sanes and Woolley 2011; Glennon et al. 2020; Persic et al. 2020). In genetic forms of congenital deafness, the critical periods do not occur normally. The functional consequences of this have been brought to light through the study of deaf patients and animals that have undergone cochlear implantation ((Peterson et al. 2010; Sharma et al. 2015) and see below). The area of the auditory cortex activated in congenitally deaf cats fitted with cochlear implants at an early age (between two and five months of age) is much larger than in deaf cats fitted with cochlear implants at an older age (more than five months). As both sets of cats were exposed to electrically evoked auditory stimulation for identical durations (Kral et al. 2002), this result strongly suggests that the plasticity revival may be less efficient after the auditory critical period. The perceptual auditory performances of humans affected by congenital deafness and fitted with cochlear implants further support this conclusion. Many studies have shown that earlier cochlear implant fitting in children is associated with better language performance (Peterson et al. 2010). Moreover, experimental paradigms based on recordings of the first component of cortical auditory-evoked potentials (CAEP), the positive peak (P1), providing information about the developmental status of the auditory cortex, and occuring at a latency of around 300 ms post-auditory stimulation in newborns and 50–70 ms in adults have yielded consistent results. In a large study, congenitally deaf children fitted with cochlear implants before the age of 3.5 years had a normal P1 latency and developed a typical invagination of the P1 wave (the N1 biomarker) with a normal timeframe, which emerges at the age of about six to seven years, whereas those fitted with implants later had an abnormally long P1 latency never reaching normal limits and rarely displayed an N1 response (Sharma et al. 2015).

All these central functional deficits are associated with multiple anatomical and structural deficits in the relay nuclei of the central auditory pathways. Studies of many models of deafness have revealed changes in synaptic structure and transmission in the auditory brainstem (Leao et al. 2006). For example, in young mutant mice lacking otoferlin (*Otof* ^−/−^ mice)—a protein acting as the calcium sensor of synaptic exocytosis in the inner hair cells (IHCs), the auditory sensory cells that make synaptic contacts with the dendrite of primary auditory neurons (Roux et al. 2006; Michalski et al. 2017)—strong synaptic deficits are also observed in the cochlear nucleus, the first compulsory central auditory synaptic relay (Wright et al. 2014). On P20, the cross sectional area of the auditory nerve and the volume of the ventral cochlear nucleus are half those in wild-type mice. Moreover, the endbulbs of Held synapses, giant synaptic terminals of the primary auditory neurons contacting the globular and spherical bushy cells in the ventral cochlear nucleus, are abnormally small, although neither the primary auditory neurons nor the ventral cochlear nucleus neurons express otoferlin. Genetic forms of congenital deafness and other forms of early auditory deprivation greatly decrease the size of the auditory cortex, with reductions in both the number of primary dendrites and the span of dendritic trees (Kral et al. 2006; Butler and Lomber 2013). Furthermore, long-range changes in connectivity involve cross-modal plasticity (Fig. 1), an adaptive reorganisation of the brain that takes place when deprivation in one sensory modality results in the recruitment of the corresponding area by other intact sensory modalities possibly compensating for sensory deficits. Such compensation was demonstrated by the enhanced visual perception of deaf cats relative to cats with normal hearing, which was eliminated by inactivating specific auditory cortical areas via direct physical cooling (Lomber et al. 2010). The existence of such changes in humans was also strongly suggested by functional magnetic resonance imaging (fRMI) findings indicating that stimuli for other sensory modalities, such as visual or tactile stimuli, activate areas of the auditory brain in adults with congenital deafness (Finney et al. 2001; Karns et al. 2012). It has recently been shown that cortical regions involved in the same type of function, such as person identification, which involves the processing of voice and face information by the auditory and visual areas, respectively, may preferentially undergo mutual reorganisation through cross-modal plasticity. Functional MRI studies have shown that regions normally involved in voice processing are preferentially reallocated to face processing, in individuals born deaf (Benetti et al. 2017). Finally, the central auditory anatomical deficits resulting from peripheral auditory deficits are not restricted to neurons and neural circuits, but also affect the cerebrovascular system. Most central auditory nuclei in the brainstem, midbrain and cortex have higher branching vessel densities than the neighbouring regions (Kirst et al. 2020), probably due to the high energy demands of sound processing in real time. Three-dimensional reconstructions of the vasculature in whole mouse brain have shown that congenital deafness (in two-month-old *Otof* ^−/−^ mice) leads to a strong decrease in vascular density in all the major relays in the auditory brainstem, midbrain and cortex (Kirst et al. 2020).

In addition to the central auditory deficits described above, which constitute an ensemble of indirect effects potentially common to all forms of congenital peripheral deafness, other indirect central deficits may be present in some specific genetic forms of congenital deafness. For instance, genetic factors and migration guidance and targeting cues control the initial coarse arrangement of the connections of auditory neuronal circuits (Cramer and Gabriele 2014; Elliott et al. 2021). The nascent neuronal networks then mature, in a process driven initially by bursts of spontaneous (i.e. independent of sensory stimuli) neuronal activity, and then by sound-evoked activity (Fig. 1). At the prehearing stage, auditory spontaneous activity is thought to be initiated by ATP release from the cochlear inner supporting cells, which activate their own purinergic receptors (P2YR1 autoreceptors). This activation results in K^+^ efflux into the extracellular space, which depolarises the IHCs and induces them to fire Ca^2+^ action potentials, which, by triggering a large influx of Ca^2+^ into the IHCs, lead to the release of the neurotransmitter, glutamate, stimulating the primary auditory neurons (Kros et al. 1998; Wang and Bergles 2015; Babola et al. 2020, 2021). This stimulation then propagates along the ascending auditory pathway, in which it is thought to calibrate the strength of synaptic relays. Spontaneous electrical activity is modulated by transient cholinergic efferent fibres, which form inhibitory synaptic contacts with IHCs (Glowatzki and Fuchs 2000; Johnson et al. 2013; Babola et al. 2021). Many cochlear cell types and molecules are involved in the generation and control of spontaneous activity. Genetic forms of deafness directly affecting the generation of spontaneous activity in the cochlea may therefore interfere with maturation of the central auditory system. For example, in mice with a knock-out of the α9 subunit of nicotinic acetylcholine receptors, subtle changes in the temporal fine structure of spontaneous activity without change in activity levels are sufficient to disturb the development of the tonotopic map in auditory brainstem nuclei (Clause et al. 2014). Abnormal spontaneous activity has also been demonstrated for genetic forms of deafness not directly involved in the generation of this activity. Indeed, afferent primary auditory neurons have been shown to have altered patterns of spontaneous activity, with specification changes, in mice with mutations of genes encoding components of the mechano-electrical transduction machinery of hair cells (Sun et al. 2018). These findings raise questions about the extent to which genetic forms of deafness affect the maturation of central auditory circuits linked to cochlear spontaneous activity and whether these central auditory deficits are reversible upon hearing restoration. The absence of spontaneous activity would be expected to have more deleterious effects for the maturation of the central auditory system than that of genetic forms affecting only the sound-evoked activity-driven steps of cochlear maturation. Within this framework, mutations of the gene encoding vesicular glutamate transporter 3, vglut3, abolishing glutamate release from IHCs should not only prevent auditory-evoked maturation of the central auditory system, but would also be expected to prevent the spontaneous activity-dependent maturation of central auditory pathways (Seal et al. 2008; Ruel et al. 2008; Babola et al. 2018). However, it is not possible to draw such a straightforward conclusion. There may be compensatory homeostatic peripheral mechanisms capable of restoring the spontaneous activity of auditory neurons, thereby preventing the most severe and early central auditory deficits. Surprisingly, in mutant mice lacking vglut3, the auditory afferent neurons display bursts of spontaneous spiking activity, albeit with an abnormal pattern relative to that in wild-type mice (Babola et al. 2018; Sun et al. 2018). The spiral ganglion neurons of the cochlea in *Vglut3*-knockout mice display enhanced excitability, facilitating direct neuronal excitation by supporting cell-induced local increases in potassium concentration, thereby bypassing the requirement for glutamate release by IHC depolarisation ((Babola et al. 2018) and see below).

Some genetic forms of deafness may also have intrinsic effects on the functioning of central auditory pathways. A substantial proportion of the genes implicated in deafness control the development or functioning of both the peripheral and central auditory systems (Willaredt et al. 2014; Michalski and Petit 2019). They encode diverse proteins, from transcription factors to ion channels, and their roles in the central auditory system are equally diverse. During early development, mutations of the genes encoding several early morphogens and transcription factors involved in the formation of both the otic placode and nearby rhombomers lead to syndromic forms of deafness affecting both the cochlea and the auditory brainstem (Michalski and Petit 2019). In some genetic forms of deafness, more subtle intrinsic central auditory deficits may be present. The detection of these deficits, which are concealed by the peripheral deficit, is likely to be particularly challenging in humans, and their consequences are, therefore, currently underappreciated, even though they may impede all attempts to restore hearing, with or without prostheses. Increasing numbers of intrinsic central auditory deficits are being discovered in animal models of genetic forms of deafness. *KCNQ4,* encoding a K^+^ channel (Kv7.4) mediating an outwardly rectifying current, was the first gene shown to play a crucial role in both the peripheral and central auditory systems (Kubisch et al. 1999; Kharkovets et al. 2000; Beisel et al. 2000). By reducing the membrane time constant, resulting in action potentials of short duration, *Kcnq4* expression is thought to ensure rapid sound processing from the periphery to the inferior colliculus. Intrinsic deficits affecting the upper levels of the auditory system may, like those of the auditory cortex, also coexist with peripheral deficits. In hair cells, the cadherin-related proteins cdhr15 and cdhr23 form the early lateral links of the hair bundle and then the tip links gating the mechanoelectrical transduction channels (Michel et al. 2005; Kazmierczak et al. 2007; Petit and Richardson 2009). An additional role for cdhr15 and cdhr23 has been identified in the embryonic mouse brain, based on the susceptibility of heterozygous *Cdhr15*^+/−^ and *Cdhr23*^+/−^ mutant mice to reflex seizures elicited by loud sounds, despite an unaffected peripheral auditory system. In the absence of either cdhr15 or cdhr23, a population of *Cdhr15*/*Cdhr23*-expressing cells derived from the medial ganglionic eminence—a major source of cortical interneuron precursors—fails to reach the auditory cortex. Moreover, later in the development of these mutant mice, parvalbumin interneurons—the most prominent class of cortical interneurons—have been shown to be present in abnormally small numbers within the auditory cortex (Libé-Philippot et al. 2017), suggesting that *Cdhr15*/*Cdhr23*-expressing cells may give rise to parvalbumin interneurons. The current challenge is to determine the extent to which the various genetic forms of deafness affecting the peripheral auditory system are associated with intrinsic central deficits, and to elucidate the effects of these deficits on central sound processing. Genes encoding proteins with key properties for the entire auditory system, such as *KCNQ4* for fast conduction, are likely to be under selection pressure to ensure broad expression across the entire sensory system. Unlike the retina and the olfactory system, the cochlea expresses virtually no specific proteins. Even prestin, a protein with piezoelectric properties involved in the cochlear amplification operated by the outer hair cells (OHCs) (Dallos et al. 2006), which was long considered to be specific to the cochlea, seems to be expressed in other tissues. We are, therefore, faced with the challenge of establishing, for proteins essential in the cochlea but with probable diverse functions, whether these proteins have been subject to positive selection for use in the central auditory system relative to other systems, especially other central sensory systems.

## Central auditory deficits in postlingual and age-related genetic forms of hearing loss

In postlingual and age-related genetic forms of hearing loss, the early phases of cochlear development mediated by deterministic genetic factors and spontaneous activity, and the successive critical periods presumably occur normally, resulting in normal early maturation of the central auditory circuits (Fig. 1). This may partly explain why the fitting of cochlear implants in profoundly deaf adults is more beneficial in patients with postlingual hearing loss than in patients with congenital hearing impairment. A scoping review on cochlear implantation outcomes found that speech perception score increased by at least 15% in 82% of adults with postlingual hearing loss but in only 54% of adults with prelingual hearing loss (Boisvert et al. 2020) fitted with implants. Many studies in animals and humans have shown that, in situations of sensory deprivation, the central auditory circuits are reorganised through maladaptive plastic changes, which may have to be reversed for future inner ear gene therapy interventions to be successful (Persic et al. 2020). In humans, one probable sign of this reorganisation is the strong relationship between tinnitus—the perception of phantom sounds—and hearing loss (Tan et al. 2013). The mechanisms underlying tinnitus are diverse and remain a matter of debate (Henry et al. 2014; Shore et al. 2016). However, hearing loss and tinnitus are frequently associated. It is widely agreed that many forms of tinnitus are associated with altered neural activity and plasticity of the central auditory system (Persic et al. 2020). Another major challenge in the health domain is deciphering the mechanisms underlying the link between mid-life hearing loss and dementia risk. People with mild, moderate or severe hearing loss in mid-life, regardless of the genetic or non-genetic nature of its cause, have a risk of developing dementia later in life that is two, three and five times higher, respectively, than that in people without hearing loss (Lin et al. 2011). The underlying biological processes remain poorly understood. Three major mechanisms have been proposed to explain this link (Uchida et al. 2019; Griffiths et al. 2020). Hearing loss and dementia may result from a common pathophysiological process independently affecting both the cochlea and the brain. The other two possible mechanisms would imply a direct relationship between peripheral hearing loss and central auditory deficits. Hearing loss may trigger a cascade of adverse events in the brain due to impoverished auditory input. Alternatively, effortful listening may mobilise cognitive resources to an unreasonable extent, at the expense of other cognitive functions, such as working memory, thereby favouring cognitive decline. In all these situations, we are still far from being able to propose possible mechanisms. However, it remains conceivable that depending on the genetic form of deafness, different mechanisms or combinations of mechanisms are involved in the link between hearing loss and dementia. Here again, the cerebrovascular system may play a key role, because its dysregulation is deeply involved in the pathophysiological processes of neurodegeneration in various forms of dementia (Wiesmann et al. 2013; Rius-Pérez et al. 2018). Generally speaking, several lines of evidence suggest that both the cochlea and central auditory system are particularly sensitive to the condition of the vascular system. One third of babies born at term following perinatal hypoxia–ischaemia have transient hearing impairment (Jiang et al. 2004). Furthermore, as mentioned above, most central auditory nuclei have higher branching vessel densities than the neighbouring regions (Kirst et al. 2020). The vascular networks of the cochlea (Nyberg et al. 2019) and central auditory system might both be affected in specific genetic forms of deafness. Alternatively, neural activity in sensory cortices has also been shown to affect the organisation of the vascular system (Whiteus et al. 2014; Lacoste et al. 2014), suggesting a tight coupling between neuronal activity and plastic remodelling of the structure of the vascular network. Consistent with this hypothesis, most of the central auditory nuclei of congenitally deaf *Otof* ^−/−^ mice have significantly lower vessel branch densities than wild-type mice at the age of two months (Kirst et al. 2020). This weakening of the cerebrovascular system following hearing loss may favour the initiation and/or progress of the pathophysiological processes associated with dementia. However, it is currently unknown whether late-onset forms of hearing loss also cause similar cerebrovascular deficits.

## Central auditory deficits associated with peripheral deficits and hearing restoration

The major issue regarding central auditory deficits in genetic forms of deafness is evaluating the extent to which they may compromise the outcome of peripheral hearing restoration. The benefits of the earlier fitting of aids before cochlear implantation in deaf children with residual hearing highlight the importance of minimising or reversing the indirect central auditory deficits linked to prolonged early sensory deprivation (Peterson et al. 2010). Cross-modal plasticity also develops over time. It improves the global sensory perception of individuals deprived of a particular sense, but is thought to be potentially detrimental for sensory restoration, in this case due to the diversion of areas of the auditory cortex away from their original sensory modality. Hypometabolism in the auditory cortex before cochlear implantation, presumably reflecting low levels of cross-modal plasticity, has been reported to be predictive of better speech performance after the fitting of cochlear implants (Lee et al. 2007). Similarly, arterial spin labelling magnetic resonance imaging (ASL-MRI) studies in deaf children recently showed that high levels of cerebral blood flow at rest before implantation in the mid- and inferior occipital areas, which are involved in language processing, were predictive of poorer speech perception after implantation, again suggesting that cross-modal reorganisation may compromise the outcome of auditory rehabilitation (Coez et al. 2021). Finding ways to control the impact of cross-modal plasticity may become a new health challenge.

Beyond the identification of central auditory deficits in the various genetic forms of deafness, it is particularly challenging to predict their reversibility, including that of deficits resulting from cross-modal plasticity. Overall, early cochlear implantation tends to result in better speech comprehension, but individual outcomes are very variable and may depend on many other factors, including residual hearing levels before implantation, post-operative rehabilitation strategies or socio-economic status (Peterson et al. 2010). In addition, cross-modal plasticity is not systematically detrimental to the efficacy of hearing restoration (Land et al. 2016). For instance, in congenitally deaf cats, the dorsal zone, an area of the secondary auditory cortex known to undergo visual cross-modal reorganisation during deafness, has been shown to restore auditory responsiveness after cochlear implantation while maintaining its response to visual stimuli demonstrating the limited detrimental effects of cross-modal plasticity on the processing of restored auditory input.

Cochlear implants are probably the most successful man–machine interface, particularly as speech perception can be achieved with a very small number of stimulating points, ~ 10–20 electrode contacts, whereas people with normal hearing make use of ~ 3000 sensory cells for fine discrimination of the full frequency representation of sounds. By contrast, cochlear gene therapies are expected to restore close-to-normal sharpness of time precision and frequency tuning in the cochlea, two essential features for challenging auditory tasks, such as speech and music processing and auditory scene analysis in noisy environments. The extent to which the untrained human auditory cortex can have cortical plasticity revived to optimise the processing of restored sensory stimulations, can partly be anticipated from studies performed on animals reared in silence, which have shown, for example, that transient auditory deprivation in gerbils achieved with ear plugs can cause persistent changes to cellular properties in the adult auditory cortex (Mowery et al. 2015). However, systematic and detailed documentation of the possible effects of a given genetic cochlear defect on other cochlear functions and on the central auditory system is often lacking. Thanks to molecular advances at the single-cell level, pathogenic processes following hearing loss can be deciphered in more detail than before. For example, in the cochlea, afferent auditory nerve fibres functionally classified into three subgroups on the basis of their firing rate at rest and their sound level-dependent activation threshold (Liberman 1978) acquire a specific transcriptomic signature over the first postnatal week in mice (Shrestha et al. 2018; Sun et al. 2018; Petitpré et al. 2018). In *Vglut3*-knockout mice, in which there is no glutamatergic release from the IHCs, the molecular specification of these subgroups of afferent auditory nerve fibres, which normally occurs between P0 and P20, is affected (Sun et al. 2018). With respect to the central auditory system, it has been shown that the distortion of sensory experience during critical periods, as in mouse pups reared in the presence of pure tones for three days upon hearing onset, is sufficient to alter gene expression patterns in auditory cortex inhibitory neurons (Kalish et al. 2020), thereby playing a key role in shaping cortical circuits during critical periods (de Villers-Sidani et al. 2008; Dorrn et al. 2010). By resolving these issues, we will be able to determine the extent to which gene therapies will need to be coupled to innovative treatments or rehabilitation for reopening plasticity windows.

There is a growing awareness that central auditory deficits must be taken into account in the management of genetic forms of peripheral deafness. One strategy for decreasing the burden of central auditory deficits on hearing restoration is preventing their build-up. For this reason, it is encouraged, in clinical practice, to fit auditory prosthetics upon hearing loss, whether prelingual or postlingual. No large-scale systematic study in humans has yet tested whether the fitting of hearing aids can help to prevent dementia, but the World Health Organisation recently recommended that governments should integrate hearing care into universal health coverage (WHO 2019). By 2040, there will be more than 80 million people suffering from dementia. Hearing restoration or rehabilitation could potentially alleviate the symptoms of dementia in more than seven million of these dementia cases (9% of cases) (Livingston et al. 2017).

Within this context, age-related hearing loss of genetic origin deserves particular attention in the development of new gene therapy strategies. It has been shown, in a large cohort of people with age-related hearing loss, that genetic predisposition to presbycusis is shaped not only by well-studied polygenic risk factors of small effect size revealed by common variants, but also by ultrarare variants probably resulting in monogenic forms. Such monogenic variants have been detected in ~ 25% of familial forms of genetic hearing loss with an onset around 50–55 years, paving the way for treatment by emerging inner ear gene therapies (Boucher et al. 2020). As age-related hearing loss occurs at a time of life at which the central auditory circuits have already been shaped, and such hearing loss is generally progressive, making it possible to preserve the auditory system by the prompt implementation of a hearing aid, these monogenic forms of age-related hearing loss may be the most promising for efficient gene therapy with limited interference from associated central auditory deficits.

## Conclusions

Thus, most genetic forms of sensorineural deafness should no longer be considered to be isolated deficits of the cochlea. Instead, they should be seen as impairments extending to the whole auditory system and beyond. Moreover, a systematic inventory of all the indirect and intrinsic central deficits associated with each particular genetic form of deafness will allow health professionals, including ENT (ear, nose and throat) specialists, SLPs (speech-language pathologists), paediatricians, and gerontologists to improve their management of hearing impairment in patients. An awareness of central deficits will be essential for the development of genuine cochlear gene therapies and to ensure scientifically-driven sensory rehabilitation before and/or after peripheral intervention.
